# Development of models for cervical cancer screening: construction in a cross-sectional population and validation in two screening cohorts in China

**DOI:** 10.1186/s12916-021-02078-2

**Published:** 2021-09-03

**Authors:** Zeni Wu, Tingyuan Li, Yongli Han, Mingyue Jiang, Yanqin Yu, Huifang Xu, Lulu Yu, Jianfeng Cui, Bin Liu, Feng Chen, Jian Yin, Xun Zhang, Qinjing Pan, Youlin Qiao, Wen Chen

**Affiliations:** 1 Department of Cancer Epidemiology, National Cancer Center/National Clinical Research Center for Cancer/Cancer Hospital, Chinese Academy of Medical Sciences and Peking Union Medical College, 17 South Panjiayuan Lane, Beijing, China; 2 Metabolic Epidemiology Branch, Division of Cancer Epidemiology & Genetics, National Cancer Institute, Bethesda, MD USA; 3 Sichuan Cancer Hospital & Institute, Sichuan Cancer Center, School of Medicine, University of Electronic Science and Technology of China, Chengdu, China; 4 Biostatistics Branch, Division of Cancer Epidemiology & Genetics, National Cancer Institute, Bethesda, MD USA; 5 Department of Public Health and Preventive Medicine, Baotou Medical College, Baotou, Inner Mongolia China; 6 Department of Cancer Epidemiology, Henan Cancer Hospital, Affiliated Cancer Hospital of Zhengzhou University, Zhengzhou, China; 7 Department of Pathology, National Cancer Center/National Clinical Research Center for Cancer/Cancer Hospital, Chinese Academy of Medical Sciences and Peking Union Medical College, 17 South Panjiayuan Lane, Beijing, China

**Keywords:** Cervical cancer, Human papillomavirus virus (HPV), Screening

## Abstract

**Background:**

Current methods for cervical cancer screening result in an increased number of referrals and unnecessary diagnostic procedures. This study aimed to develop and evaluate a more accurate model for cervical cancer screening.

**Methods:**

Multiple predictors including age, cytology, high-risk human papillomavirus (hrHPV) DNA/mRNA, E6 oncoprotein, HPV genotyping, and p16/Ki-67 were used for model construction in a cross-sectional population including women with normal cervix (*N* = 1085), cervical intraepithelial neoplasia (CIN, *N* = 279), and cervical cancer (*N* = 551) to predict CIN2+ or CIN3+. A base model using age, cytology, and hrHPV was calculated, and extended versions with additional biomarkers were considered. External validations in two screening cohorts with 3-year follow-up were further conducted (*N*_Cohort-I_ = 3179, *N*_Cohort-II_ = 3082).

**Results:**

The base model increased the area under the curve (AUC, 0.91, 95% confidence interval [CI] = 0.88–0.93) and reduced colposcopy referral rates (42.76%, 95% CI = 38.67–46.92) compared to hrHPV and cytology co-testing in the cross-sectional population (AUC 0.80, 95% CI = 0.79–0.82, referrals rates 61.62, 95% CI = 59.4–63.8) to predict CIN2+. The AUC further improved when HPV genotyping and/or E6 oncoprotein were included in the base model. External validation in two screening cohorts further demonstrated that our models had better clinical performances than routine screening methods, yielded AUCs of 0.92 (95% CI = 0.91–0.93) and 0.94 (95% CI = 0.91–0.97) to predict CIN2+ and referrals rates of 17.55% (95% CI = 16.24–18.92) and 7.40% (95% CI = 6.50–8.38) in screening cohort I and II, respectively. Similar results were observed for CIN3+ prediction.

**Conclusions:**

Compared to routine screening methods, our model using current cervical screening indicators can improve the clinical performance and reduce referral rates.

**Supplementary Information:**

The online version contains supplementary material available at 10.1186/s12916-021-02078-2.

## Background

Cervical cancer is the fourth most frequently diagnosed cancer and the fourth leading cause of cancer death in women, with an estimated 570,000 new cases and 311,000 deaths in 2018 worldwide [1]. Cancer morbidity and mortality have decreased in developed countries due to the implementation of routine cervical cancer screening [2], and testing for high-risk human papillomavirus (hrHPV) has improved cervical cancer prevention efforts [3].

The decision for modern cervical cancer screening programs is often made based on age, cytology, and hrHPV testing results. For example, the USA has different cervical cancer screening guidelines for women in different age groups. For women aged 30 to 65 years, guidelines recommend the use of cytology and hrHPV co-testing due to its high sensitivity. HPV testing has not been recommended in women aged 21–29 without atypical squamous cells of undetermined significance (ASC-US) due to its low specificity [4]. However, the cytology and hrHPV co-testing would increase the number of referrals, unnecessary diagnostic procedures, and costs of the health care system [5, 6].

Cervical intraepithelial neoplasia grade 2/3 (CIN2/3) can progress to cervical cancer if left untreated, and therefore identifying women who would benefit from further monitoring and/or treatment is important [7]. However, we need to ensure that unnecessary referrals are avoided. To retain the high sensitivity of our current primary screening tests and improve the specificity, additional screening biomarkers such as HPV genotyping [8, 9], E6 oncoprotein [10], or p16/Ki-67dual staining [11] have been developed and shown to have high specificity as triage tests for HPV positive women, respectively. However, decision-making in routine cancer screening may become more complicated as additional biomarkers are added and screening algorithms become increasingly complex. These complex screening algorithms may have limited practical applications.

In recent years, machine learning methods play an important role in selecting an appropriate combination of multiple biomarkers. With the increasing availability of large national databases and computing power, the use of machine learning methods in medical science and health care has been rapidly growing [12, 13]. Studies have shown that machine learning methods such as logistic regression and support vector machine (SVM) can enhance prediction performances by providing clinicians with valuable evidence-based prognostic information. By using the machine learning methods, we may substantially improve the sensitivity and specificity of cervical cancer screening, avoiding unnecessary colposcopy referral, and simplifying decision-making in clinical practice.

The aim of this study was to develop models that have better prediction of CIN2+ by using age, cytology, and hrHPV testing, with or without other biomarkers. Models were constructed and evaluated in a cross-sectional population enriched with CIN and cervical cancer. External validation in two screening cohorts was further presented to demonstrate the usefulness of our methods.

## Methods

### Study population

**Fig. 1:**
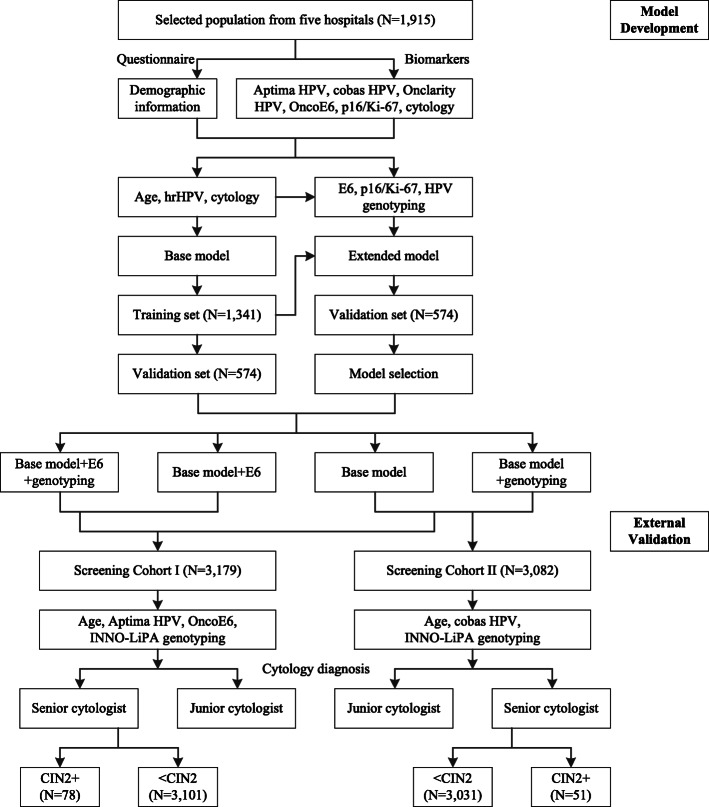
Flow chart of the study

This study included three populations, one cross-sectional population and two screening cohorts. Women were eligible if they had an intact cervix and no prior history of CIN. Women eligible for the screening cohorts were additionally aged 25 to 65. Women who were pregnant, had a hysterectomy, or received treatment for cervical diseases were excluded. Further details on the study design are provided in Fig. 1. Institutional review board (IRB) approval was provided by the Ethics Committee from Cancer Hospital, Chinese Academy of Medical Sciences. All participants have agreed on the study protocol and provided informed consent.

#### Cross-sectional population

Participants were recruited from five hospitals in China between 2014 and 2015 and included women attending routine cervical cancer screening programs, outpatients referred for colposcopy, and inpatients planning treatment for CIN2+. A questionnaire was used to collect information on demographic factors and obstetrics and gynecology history. Two cervical exfoliated cell samples were collected: one was kept in PreservCyt Solution (Hologic) and aliquoted for cobas HPV (Roche), Aptima HPV (Hologic), Onclarity HPV (BD Diagnostics) testing, p16/Ki-67 dual staining (Roche), and liquid-based cytology (LBC) assessment and the other sample was kept in a Dacron swab for HPV16/18 E6 protein detection (Arbor Vita Corporation). Cervical biopsies were conducted using a protocol as previously described [14]. Local pathologists provided the primary diagnosis, and a panel of five pathologists from each center underwent a diagnostic blind review for consensus.

#### Screening cohorts

Both screening cohorts included a baseline phase and a 3-year follow-up phase. Participants in the screening cohort I (SC-I) were recruited from Shanxi Province of China between 2017 and 2020. At baseline, all participants received Aptima HPV, INNO-LiPA HPV genotyping (Innogenetics), and LBC. Aptima HPV positive samples were tested by Aptima HPV16/18/45. Women with HPV16/18/45 positive or abnormal cervical cytology (ASC-US+) were referred for colposcopy and women with HPV16/18/45 results had an additional swab for E6 oncoprotein test collected before colposcopy.

Participants in the screening cohort II (SC-II) were recruited from the Inner Mongolia Autonomous Region of China between 2016 and 2019. At baseline, all participants received cobas HPV, INNO-LiPA HPV genotyping, and LBC. Women with HPV16/18 positive or ASC-US+ were referred for colposcopy.

For both screening cohorts, women who were HPV positive or had an ASC-US+ cytology continued to annual follow-up visits, and all women regardless the results at baseline came back at the 3rd year for a final visit. At each visit, a LBC specimen was obtained and women with ASC-US+ were referred for colposcopy. Women found to have a diagnosis of CIN2+ at baseline or follow-up exited the study after the colposcopy visit and were referred for treatment.

### Laboratory tests

The Onclarity HPV is a PCR assay for the detection of six individual HPV genotypes (16, 18, 31, 45, 51, and 52) and three groups of types (33/58, 59/56/66, and 39/68/35). The cobas HPV is another PCR assay for the detection of viral DNA of the 14 hrHPV types, which simultaneously differentiates HPV16 and HPV18. The Aptima HPV is based on the qualitative detection of E6/E7 mRNA of 14 hrHPV types. The Aptima HPV16/18/45 uses the same technology as Aptima HPV for detection of E6/E7 mRNA from HPV16/18/45; the assay differentiates genotype 16 from 18 and 45 but does not differentiate between 18 and 45. INNO-LiPA HPV genotyping assay allows simultaneous and separate detection of 25 different HPV genotypes (14 hrHPV and HPV6, 11, 34, 40, 42, 43, 44, 53, 54, 70, and 74). All HPV tests were performed at the fully automated system according to the manufacturer’s instructions. The OncoE6 cervical test is an immunochromatographic test for the detection of HPV16/18 E6 oncoprotein. The operation procedures were described previously [15].

Cytology slides were first evaluated by junior cytologists and then diagnosed by senior cytologists. Results were reported using the Bethesda 2014 nomenclature. A second cytology slide was prepared from the residual PreservCyt Solution for p16/Ki-67 dual staining using the CINtecPLUS Cytology kit according to the manufacturer’s instructions for the cross-sectional samples. Technicians were blinded to each other’s findings to minimize bias.

### Statistical analyses

#### Model development

Based models of logistic regression and SVM were implemented on the platform of R (Version 3.5.2). Model construction and internal validation were performed in the cross-sectional population, which was randomly split into 70% for a training set and 30% for a testing set.

Logistic regression or SVM using age, cytology, and hrHPV as predictors was set as the base model. Among the predictors, age was a continuous covariate; hrHPV testing was dichotomous (any type of the 14 hrHPV types positive vs. all of the 14 hrHPV types negative); and cytology was a seven-level covariate: negative for intraepithelial lesion or malignancy (NILM), ASC-US, low-grade squamous intraepithelial lesion (LSIL), atypical squamous cells cannot exclude high-grade lesion (ASC-H), atypical glandular cell (AGC), high-grade squamous intraepithelial lesion/adenocarcinoma in situ (HSIL/AIS), and squamous cell carcinoma/adenocarcinoma (SCC/ADC). HSIL and AIS, as well as SCC and ADC, were separately combined because limited cases were available for these levels. CIN2+ or CIN3+, the outcome of interest, was dichotomous. Receiver operating characteristic (ROC) curve (sensitivity and 1-specificity) and the area under the curve (AUC) were used to assess predictive accuracy. Sensitivity, specificity, and colposcopy referral rate were also calculated for current screening methods and models based on the thresholds with the largest Youden Index.

The base model was extended by substituting hrHPV using different detection methods, i.e., the result of cobas was substituted by Aptima or Onclarity. Additional covariates were also added to the base model, including E6 oncoprotein (dichotomous, either HPV16/18 positive vs. both HPV16&18 negative), p16/Ki-67 (dichotomous, positive vs. negative), and HPV genotyping (nine dummy variables: HPV16, 18, 31, 45, 51, 52, 33/58, 59/56/66, and 39/68/35, positive vs. negative). AUCs were compared using the “pROC” package in R. Logistic regression or SVM, which one showed better clinical performance, was chosen for further analysis. Statistical significance was assessed by two-tailed tests with *α* level of 0.05.

#### External validation in screening cohorts

The base model and extended versions with HPV genotyping were applied to both screening cohorts. The extended models with E6 oncoprotein were applied to SC-I only because swab samples were not collected in SC-II. Cytology results diagnosed by junior and senior cytologists were also evaluated in models. Three-year cumulative risks of CIN2+ were estimated by hrHPV and cytology co-testing negative and predicted-negative populations.

## Results

### Study population characteristics

Table 1 shows the characteristics of the study populations. A total of 1915, 3179, and 3082 women were eligible in the cross-sectional population, SC-I, and SC-II, respectively. The average ages (years ± standard deviation) of women were 47.79±9.78, 45.22±7.76, and 42.80±8.85; the positivity rates of HPV were 50.81%, 13.90%, and 17.07%; the abnormal cytology proportions were 53.16%, 10.47%, and 17.46%; and the CIN2+ percentages were 39.06%, 2.45%, and 1.65%, respectively.

**Table 1:** Characteristics of three study populations at baseline

	Cross-sectional population (***N*** = 1915)	Screening cohort I (***N*** = 3179)	Screening cohort II (***N*** = 3082)
**Age (mean±SD)**	47.79±9.78	45.22±7.76	42.80±8.85
**HPV (%)**			
Positive	973 (50.81)	442 (13.90)	526 (17.07)
HPV16/18 positive	639 (33.42)	126 (3.96)	155 (5.03)
Negative	940 (49.09)	2,737 (86.10)	2,556 (82.93)
NA	2 (0.10)	0 (0.00)	0 (0.00)
**Cytology (%)**			
NILM	897 (46.84)	2846 (89.53)	2544 (82.54)
ASC-US	222 (11.59)	154 (4.84)	330 (10.71)
ASC-H	81 (4.23)	17 (0.53)	27 (0.88)
AGC	14 (0.73)	5 (0.16)	14 (0.45)
LSIL	103 (5.38)	91 (2.86)	121 (3.93)
HSIL/AIS	242 (12.64)	62 (1.95)	39 (1.27)
SCC/ADC	356 (18.59)	4 (0.13)	7 (0.23)
**Pathology (%)**			
No history of CIN	1085 (56.66)	3039 (95.60)	2996 (97.21)
CIN1	82 (4.28)	62 (1.95)	35 (1.14)
CIN2	61 (3.19)	35 (1.10)	28 (0.91)
CIN3	136 (7.10)	39 (1.23)	21 (0.68)
SCC	506 (26.42)	3 (0.09)	2 (0.06)
ADC	45 (2.35)	1 (0.03)	0 (0.00)

*SD* standard deviation

### Model development

**Fig. 2:**
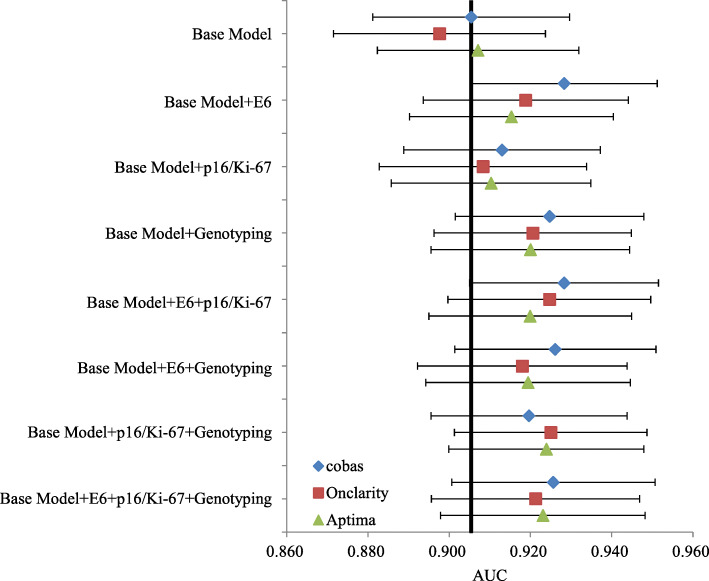
Area under the receiver operating characteristic curve (AUC) and 95% confidence interval (CI) of the base model with or without additional biomarkers

Results for the current screening methods and the proposed models for CIN2+ prediction are presented in Table 2. Statistical comparisons showed that the logistic regression had slightly higher AUC compared to SVM thus were chosen in further analysis (parameters of the models are shown in Additional file 1: Table S1-S2). The logistic regression from the testing set of the cross-sectional population showed that the base model had a sensitivity of 92.00% (95% confidence interval [CI] = 88.00–95.11%), specificity of 89.08% (95% CI = 85.63–92.24%), and AUC of 0.91 (95% CI = 0.88–0.93). The AUC of the base model slightly increased when p16/Ki-67 dual staining was added in the base model, whereas larger AUC improvements were obtained when HPV genotyping or E6 oncoprotein were included in the base model (Fig. 2). Results of cobas, Aptima, and Onclarity showed no significant changes.

**Table 2:** Clinical performance of current screening methods and models for cross-sectional population (CIN2+)

		Sensitivity % (95% CI)	Specificity % (95% CI)	AUC (95% CI)	Referral rate % (95% CI)
**Cross-sectional population (*N*= 1915)**					
Current methods in testing set	hrHPV DNA (cobas)	92.49 (90.62–94.37)	75.75 (73.26–78.23)	0.84 (0.83–0.86)	50.86 (48.60–53.13)
	hrHPV mRNA (Aptima)	93.68 (91.94–95.43)	81.29 (79.11–83.57)	0.87 (0.86–0.89)	48.25 (45.98–50.53)
	hrHPV DNA (Onclarity)	91.36 (89.37–93.34)	78.24 (75.78–80.79)	0.85 (0.83–0.86)	49.63 (47.27–51.99)
	HPV16/18 DNA (cobas)	75.44 (72.35–78.52)	93.40 (91.86–94.77)	0.84 (0.83–0.86)	33.42 (31.31–35.59)
	HPV16/18 E6	66.52 (62.84–69.93)	97.70 (96.70–98.60)	0.82 (0.80–0.84)	28.84 (26.70–31.05)
	p16/Ki-67	85.07 (82.33–87.67)	79.41 (76.89–81.67)	0.82 (0.80–0.84)	45.67 (43.40–47.95)
	ASC-US+	95.99 (94.52–97.33)	74.38 (71.72–76.78)	0.85 (0.84–0.87)	53.16 (50.89–55.41)
	Co-testing	98.40 (97.33–99.20)	62.04 (59.13–64.70)	0.80 (0.79–0.82)	61.62 (59.40–63.80)
**Cross-sectional population validation set (*N*= 575)**					
Logistic regression	Base model	92.00 (88.00–95.11)	89.08 (85.63–92.24)	0.91 (0.88–0.93)	42.76 (38.67–46.92)
	Base model + E6	92.49 (88.73–95.77)	93.17 (90.44–95.90)	0.93 (0.91–0.95)	42.89 (38.53–47.33)
	Base model + GT	92.79 (89.42–96.15)	92.16 (89.22–95.10)	0.92 (0.90–0.95)	42.22 (37.91–46.62)
	Base model + E6 + GT	90.31 (86.22–94.39)	94.92 (92.19–97.27)	0.93 (0.90–0.95)	42.04 (37.44–46.74)
Support vector machine	Base model	87.11 (82.22–91.56)	92.24 (89.37–94.83)	0.90 (0.87–0.92)	38.92 (34.90–43.05)
	Base model + E6	92.96 (89.20–96.24)	88.40 (84.98–91.81)	0.91 (0.88–0.93)	45.85 (41.44–50.30)
	Base model + GT	87.02 (82.21–91.35)	91.18 (87.91–94.12)	0.89 (0.86–0.92)	40.47 (36.19–44.85)
	Base model + E6 + GT	88.78 (84.18–92.86)	91.80 (88.28–94.92)	0.90 (0.88–0.93)	43.14 (38.52–47.85)

*AUC* area under the curve, *E6* E6 oncoprotein, *GT* HPV genotyping

For the current screening methods, the largest AUCs were obtained by ASC-US+ (AUC = 0.85, 95% CI=0.84–0.87) and HPV mRNA (AUC = 0.87, 95% CI = 0.86–0.89). For co-testing, the AUC was 0.80 (95% CI = 0.79–0.82). The base model showed to have slightly higher AUC compared to current screening methods using either ASC-US+ or hrHPV mRNA. In addition, the base model reduced the number of colposcopy referrals, with a referral rate of 42.76% (95% CI = 38.67–46.92%) compared to 61.62% (95% CI = 59.40–63.80%) by hrHPV and cytology co-testing, 48.25% (95% CI = 45.98–50.53%) by hrHPV mRNA, and 53.16% (95% CI = 50.89–55.41%) by ASC-US+. The referral rates of the base model were further reduced when additional predictors were used (Table 2).

Similar results for the current screening methods and the proposed models for CIN3+ prediction are presented in Additional file 1: Table S3.

### External validation in screening cohorts at baseline

The models were further applied to the baseline data of the two screening cohorts, with or without E6 oncoprotein and/or HPV genotyping for CIN2+ (Table 3) and CIN3+ (Additional file 1: Table S3) prediction. For CIN2+ prediction, the base models of SC-I and SC-II yielded sensitivities of 100% and 94.12% (95% CI = 86.27–100.00%), specificities of 0.84.49% (95% CI = 83.23–85.75%) and 94.06% (95% CI = 93.20–94.89%), and AUCs of 0.92 (95% CI = 0.91–0.93) and 0.94 (95% CI=0.91–0.97), respectively, better than hrHPV and cytology co-testing. The base model also had lower colposcopy referral rates than co-testing (17.55%, 95% CI = 16.24–18.92%, versus 20.64% 95% CI = 19.24–22.08% in SC-I; and 7.40% 95% CI = 6.50–8.38%, versus 26.83% 95% CI = 25.28–28.44% in SC-II). Although the models based on the diagnosis of junior cytologists did not perform as well as those using the diagnosis from senior cytologists, their AUCs were still higher than the corresponding hrHPV and cytology co-testing in both cohorts. The inclusion of E6 oncoprotein and/or HPV genotyping into the base model slightly increased AUCs in the baseline data. Similar results were observed using CIN3+ as the outcome.

**Table 3:** Clinical performance of current screening methods and models for screening cohorts at baseline (CIN2+)

		Sensitivity % (95% CI)	Specificity % (95% CI)	AUC (95% CI)	Referral rate % (95% CI)
**Screening cohort I (*N*= 3179)**					
HPV	hrHPV mRNA	97.44 (93.59–100.00)	85.94 (84.65–87.20)	0.92 (0.90–0.94)	16.14 (14.87–17.46)
Senior cytologists	ASC-US	87.18 (79.49–93.59)	91.45 (90.52–92.39)	0.89 (0.86–0.93)	10.47 (9.43–11.59)
	Co-testing	100.00	81.39 (80.01–82.75)	0.91 (0.90–0.91)	20.64 (19.24–22.08)
	Base model	100.00	84.49 (83.23–85.75)	0.92 (0.92–0.93)	17.55 (16.24–18.92)
	Base model + E6	93.59 (87.18–98.72)	92.26 (91.29–93.20)	0.93 (0.90–0.96)	9.81 (8.80–10.90)
	Base model + GT	97.44 (93.59–100.00)	89.39 (88.26–90.42)	0.93 (0.92–0.95)	12.77 (11.63–13.98)
	Base model + E6 + GT	97.44 (93.59–100.00)	89.04 (87.91–90.07)	0.93 (0.91–0.95)	13.09 (11.93–14.31)
Junior cytologists	ASC-US	85.90 (78.21–93.59)	87.39 (86.20–88.55)	0.87 (0.83–0.91)	14.38 (13.17–15.64)
	Co-testing	100.00	77.91 (76.46–79.26)	0.89 (0.88–0.90)	24.03 (22.56–25.56)
	Base model	100.00	83.49 (82.20–84.78)	0.92 (0.91–0.92)	18.56 (17.22–19.96)
	Base model + E6	91.03 (84.62–96.19)	91.97 (91.04–92.94)	0.92 (0.88–0.95)	10.07 (9.04–11.16)
	Base model + GT	96.15 (91.03–100.00)	88.39 (87.23–89.49)	0.92 (0.90–0.94)	13.68 (12.51–14.93)
	Base model + E6 + GT	96.15 (91.03–100.00)	87.81 (86.62–88.94)	0.92 (0.90–0.94)	14.22 (13.02–15.48)
**Screening cohort II (*N*= 3082)**					
HPV	hrHPV DNA	90.20 (82.30–98.04)	84.16 (82.88–85.42)	0.87 (0.83–0.91)	17.07 (15.75–18.44)
Senior cytologists	ASC-US	96.08 (90.20–100.00)	83.90 (82.55–85.19)	0.90 (0.87–0.93)	17.46 (16.13–18.84)
	Co-testing	100.00	74.40 (72.81–76.01)	0.87 (0.86–0.88)	26.83 (25.28–28.44)
	Base model	94.12 (86.27–100.00)	94.06 (93.20–94.89)	0.94 (0.91–0.97)	7.40 (6.50–8.38)
	Base model + GT	96.08 (90.20–100.00)	95.17 (94.34–95.93)	0.96 (0.93–0.98)	6.33 (5.49–7.25)
Junior cytologists	ASC-US	90.20 (80.39–98.04)	80.53 (79.18–81.95)	0.85 (0.81–0.90)	20.64 (19.22–22.11)
	Co-testing	98.04 (94.12–100.00)	71.16 (69.55–72.75)	0.85 (0.83–0.87)	29.98 (28.37–31.63)
	Base model	88.24 (78.43–96.08)	90.76 (89.74–91.82)	0.90 (0.85–0.94)	10.55 (9.48–11.68)
	Base Model+GT	98.04 (94.12–100.00)	89.64 (88.55–90.70)	0.94 (0.92–0.96)	11.78 (10.66–12.97)

AUC area under the curve, *E6* E6 oncoprotein, *GT* HPV genotyping

### External validation in screening cohorts at follow-up

During the 3-year follow-up procedures, 42 CIN2+ cases were diagnosed in SC-I, with 37 cases predicted to be positive and 5 cases to be negative by base model at baseline. These 5 cases were both hrHPV negative and normal cytology at baseline. Women with predicted-negative findings had slightly lower 3-year risks of CIN2+ compared with women with hrHPV and cytology co-test negative (0.19%, 95% CI = 0.06–0.44% vs 0.20%, 95% CI = 0.06–0.46%). As for SC-II, 28 CIN2+ cases were diagnosed during follow-up, with 11 cases predicted to be positive and 17 cases to be negative at baseline. Women with predicted-negative findings had higher 3-year risks of CIN2+ compared with women with co-test negative (0.70%, 95% CI = 0.43–1.08% vs 0.09%, 95% CI = 0.01–0.32%).

Since the 3-year risk of CIN2+ was higher among women with negative results of the predictive model compared to co-testing, we changed the thresholds from the highest Youden Index to the highest sensitivity for base model prediction. Results for SC-I did not change, whereas SC-II yielded a sensitivity of 100%, specificity of 84.13% (95% CI = 82.88–85.42%), and colposcopy referral rate of 17.23% (95% CI = 15.91–18.61%), which also has a higher specificity and AUC, same sensitivity and lower colposcopy referral rate compared to co-testing at baseline. By using this threshold, 26 out of 28 follow-up CIN2+ cases were predicted to be positive at baseline and 2 cases were negative. Women with predicted-negative findings had slightly lower 3-year risks of CIN2+ (0.08%, 95% CI = 0.01–0.28%) compared with 0.09% (95% CI = 0.01–0.32%) of women with co-testing negative.

## Discussion

In this study, we developed and evaluated machine learning-based models to predict CIN2+ or CIN3+ for cervical cancer screening. A logistic regression model using hrHPV, cytology, and age was set as the base model due to its superior performances in prediction and colposcopy referral rates reduction. Improved clinical performance of the base model can be gained by incorporating E6 oncoprotein and/or HPV genotyping information. External validation in two screening cohorts further demonstrated that our models had better clinical performances than routine screening methods. The 3-year risks of CIN2+ for the predicted-negative women depended on the thresholds of the model, but the improvement of clinical performance at baseline can be obtained whichever threshold was chosen.

Different models were used for cervical cancer screening in previous studies. Karakitsos et al. used the learning vector quantizer neural network classifier on cytological diagnosis, HPV DNA test, E6/E7 HPV mRNA test, and p16 immunostaining to build an algorithm to facilitate the classification of CIN2+. This model improved the AUC (0.916) significantly compared to cytology diagnosis alone (0.866) [16]. In the study conducted by Branca et al., comprehensive multivariate models were constructed by a panel of 13 biomarkers to predict CIN2+, giving the AUC of 0.897 [17]. A Korean study developed a web-based tool on age, cytology and presence of 15 hrHPV genotypes in a SVM model to identify the patient features that maximally contributed to progression to cervical lesions, which obtained an accuracy of 74.41%. However, this model was not developed for cancer screening and their result was highly dependent on the proportion of positive and negative individuals they selected [18]. Several studies used logistic regression to establish predictors for histologic grade or risk stratification based on the epidemiologic risk factors and the molecular markers. In a large study of around 100,000 women using race, smoking status, insurance, marital status, median income, and previous HPV test result as predictors, their model only obtained an AUC of 0.81 for CIN2+ [19]. Another study of 1,477 women reported that the most predictive factors were mRNA level, DNA index, parity, and age, and the AUC was 0.99 for HSIL and 0.81 for LSIL [20]. However, findings from previous studies may not be replicable across studies due to differences in adjustment factors, sample size, and degrees of diagnoses.

The clinical performances of our extended models included HPV genotyping and/or E6 oncoprotein showed to be better than the base model in each of the study population assessed, but resulted in a slight increase in cost. HPV genotyping can be a byproduct of HPV testing that has little additional costs but more additional information. E6 oncoprotein is pivotal in initiation and maintenance of oncogenic transformation by HPV [21] and associated with viral persistence [22, 23]. The protein testing is a lateral flow immunoassay designed for low- and middle-income countries [15]. When conducting study in SC-I, we assumed that only people positive with HPV mRNA result could express E6 oncoprotein. Therefore, our protein testing was performed only in the HPV16/18/45 mRNA-positive participants (*N* = 126). These results showed that additionally testing for the E6 oncoprotein in a limited group of people could yield better screening performance than the base model. In addition, the models recommended fewer women to receive immediate colposcopy compared to HPV and cytology testing alone or co-testing but had the same cost with co-testing in the real-world setting to collect HPV testing and cytology information, hence could reduce unnecessary diagnostic procedures and costs.

Cytology diagnosis is subjective in nature, and its reproducibility and accuracy are affected by the cytologist’s skill [24]. In our study, the cytology results diagnosed by senior cytologists were performed in a high-quality laboratory in Beijing, which may not be generalizable to all the cytology laboratories [25]. For example, the cytology diagnosis in SC-II was conducted by the best cytologist in China, whose sensitivity (0.961) was higher than HPV testing (0.902). In order to better extrapolate, models based on the cytology results from junior cytologists were also evaluated. Although we found that model performances were affected by the skill level of the cytologists, the clinical performances of our models were still increased, compared to hrHPV testing and cytology co-testing within the same cytologist’s skill level.

## Conclusions

Our study demonstrated that machine learning could incorporate multiple screening methods into one algorithm and develop models by the current cervical cancer screening indicators, which has the potential to be a reliable screening method considering its better clinical performance and lower referral rate.

## Supplementary Information

**Additional File 1:Table S1-S3.Table S1.** – Logistic Regression Parameters. **Table S2.** – SVM Parameters. **Table S3.** – Clinical performance of current screening methods and models for cross-sectional population and screening cohorts at baseline (CIN3+)

## Data Availability

The datasets used and/or analyzed during the current study are available from the corresponding author on reasonable request.

## Abbreviations

ADC: Adenocarcinoma
AGC: Atypical glandular cell
AIS: Adenocarcinoma in situ
ASC-H: Atypical squamous cells cannot exclude high-grade lesion
ASC-US: Atypical squamous cells of undetermined significance
AUC: The area under the curve
CI: Confidence interval
CIN: Cervical intraepithelial neoplasia
HPV: Human papillomavirus virus
HSIL: High-grade squamous intraepithelial lesion
LBC: Liquid-based cytology
LSIL: Low-grade squamous intraepithelial lesion
NILM: Negative for intraepithelial lesion or malignancy
ROC: Receiver operating characteristic
SCC: Squamous cell carcinoma
SC-I: Screening cohort I
SC-II: Screening cohort II
SVM: Support vector machine
